# Potential biomarkers for predicting immune response and outcomes in lung cancer patients undergoing thermal ablation

**DOI:** 10.3389/fimmu.2023.1268331

**Published:** 2023-11-01

**Authors:** Jing Sang, Xin Ye

**Affiliations:** Department of Oncology, The First Affiliated Hospital of Shandong First Medical University & Shandong Provincial Qianfoshan Hospital, Shandong Lung Cancer Institute, Shandong Key Laboratory of Rheumatic Disease and Translational Medicine, Jinan, China

**Keywords:** lung cancer, thermal ablation, biomarkers, immune response, outcome

## Abstract

Thermal ablation is a promising alternative treatment for lung cancer. It disintegrates cancer cells and releases antigens, followed by the remodeling of local tumor immune microenvironment and the activation of anti-tumor immune responses, enhancing the overall effectiveness of the treatment. Biomarkers can offer insights into the patient’s immune response and outcomes, such as local tumor control, recurrence, overall survival, and progression-free survival. Identifying and validating such biomarkers can significantly impact clinical decision-making, leading to personalized treatment strategies and improved patient outcomes. This review provides a comprehensive overview of the current state of research on potential biomarkers for predicting immune response and outcomes in lung cancer patients undergoing thermal ablation, including their potential role in lung cancer management, and the challenges and future directions.

## 1 Introduction

Lung cancer, with non-small cell lung cancer (NSCLC) constituting 80-85% of all cases, is a principal global cause of cancer-related mortality (1). While advancements in early detection and therapies like immunotherapies and targeted interventions have been achieved, the overall survival rates are still suboptimal, especially for those with advanced or metastatic conditions (2). For long, the cornerstone of lung cancer management has been traditional modalities such as surgery, chemotherapy, and radiation therapy (3). Yet, a burgeoning interest in minimally invasive methods like thermal ablation is evident, offering potential for superior outcomes and enhanced quality of life. These approaches are particularly beneficial for patients ineligible for standard treatments or those grappling with recurrent or oligometastatic disease (4, 5).

Emerging as promising substitutes for surgery and other localized treatments, thermal ablation techniques such as radiofrequency ablation (RFA), microwave ablation (MWA), cryoablation, laser ablation, and high intensity focused ultrasound (HIFU) are proving beneficial for various cancers (6–9). By applying extreme temperatures (heat or cold), these methods induce localized tumor cell death while minimizing damage to surrounding healthy tissue. Critical to the success of thermal ablation is its capacity to influence the tumor immune microenvironment (TIME), which significantly impacts tumor progression, metastasis, and therapy responsiveness (10).

TIME consisting of diverse immune cells, cytokines, chemokines, and other molecules, can both accelerate and inhibit tumor growth and metastasis (11). Thermal ablation’s influence on TIME can trigger anti-tumor immune responses, thereby boosting the overall efficacy of the treatment (12, 13). However, patients’ responses to thermal ablation can differ significantly due to variations in technique and associated technical parameters. Therefore, identifying predictive biomarkers becomes essential to select those most likely to benefit from this therapeutic approach.

Biomarkers can provide valuable information on the patient’s immune status, tumor characteristics, and potential response to therapy. In the context of lung cancer thermal ablation, predictive biomarkers can offer insights into the patient’s immune response and treatment outcomes, such as local tumor control, recurrence, overall survival (OS), and progression-free survival (PFS). Identifying and validating such biomarkers can significantly impact clinical decision-making, leading to personalized treatment strategies and improved patient outcomes.

This review aims to provide a comprehensive overview of the current state of research on potential biomarkers for predicting immune response and outcomes in lung cancer patients undergoing thermal ablation. We will discuss various types of biomarkers, their potential role in lung cancer management, and the challenges and future directions in this rapidly evolving field.

## 2 Thermal ablation techniques in lung cancer

### 2.1 Overview of various thermal ablation techniques

Thermal ablation techniques have emerged as a valuable addition to the arsenal of lung cancer treatments, particularly for patients who may not be ideal candidates for surgery or those grappling with recurrent or oligometastatic disease (14). The most prevalent thermal ablation techniques employed in managing lung cancer include RFA, MWA and cryoablation.

RFA, the earliest technique employed for treating solid tumors, hinges on inserting an electrode into the tumor tissue. Under the influence of a high-frequency alternating current (375-500 kHz), the ions within the tumor tissue generate thermal biological effects due to friction and collision. The local temperature can soar to 60°C-120°C, resulting in coagulative necrosis of cells when heated above 60°C. The volume of ablated tissue through RFA is determined by the local heat conduction generated by RFA, alongside the heat convection between circulating blood and extracellular fluid (15). For lesions that are in proximity to large blood vessels and airways, the effectiveness of ablation may be reduced due to the phenomenon of ‘heat sink’, which is the dissipation of heat by adjacent vessels through convection. RFA is a safe and effective treatment that provides a survival benefit for selected patients with primary and secondary lung tumors (16).

MWA harnesses electromagnetic waves to create heat, it generally adopts two frequencies, 915-MHz or 2,450-MHz. In the presence of microwave electromagnetic fields, polar molecules like water and proteins within tumor tissues exhibit extremely high-speed vibrations. These movements instigate molecular collision and friction, leading to a rapid surge in temperature up to 60°C-150°C, thereby inducing coagulative necrosis within the tumor. Moreover, MWA has the advantages of high-convective and low-heat deposition effect in the lung (17). Therefore, MWA allows for shorter ablation times and larger ablation volumes compared to RFA (18). Recent studies have documented encouraging results with MWA in terms of safety, efficacy, and local tumor control among lung cancer patients (19).

Cryoablation techniques comprise argon-helium cryoablation and liquid nitrogen cryoablation. (1) Argon-helium cryoablation, a more established method, leverages the Joule-Thomson effect. High-pressure argon cools the target tissue to -140°C, while helium rapidly warms it back to -20°C-40°C. (2) Liquid nitrogen cryoablation cools the target tissue to -196°C, using ethanol to release significant heat during its vaporization to the liquefaction state, warming the tissue above 80°C (20). These shifts in temperature gradient from cryoablation can induce protein denaturation, cell lysis due to altered osmotic pressure and the “icing” effect, tissue ischemia and necrosis from microembolization, and the release of tumor antigens that incite anti-tumor immunity (21, 22). Cryoablation is a more time-consuming procedure than RFA and MWA. However, it has several advantages such as easy visualization of the ice ball on CT, preservation of collagenous architecture, and less intraprocedural pain due to the anesthetic effects of cold. Cryoablation may be more suitable for central tumors close to the tracheobronchial tree and peripheral subpleural lesions treated without general anesthetic (23). Cryoablation is a safe and feasible treatment for malignant lung tumors, with acceptable rates of adverse events (24).

In conclusion, thermal ablation, an independent local tumor treatment technology, has emerged as the third principal local therapeutic strategy for tumors, after surgery and stereotactic body radiation therapy (SBRT). Its use in comprehensive lung tumor treatment is poised to expand. Yet, certain limitations exist when applying thermal ablation techniques to lung tumors. The potential of integrating thermal ablation with molecularly targeted drug therapy and immunotherapy has been explored and warrants further research (25, 26).

### 2.2 Impact of thermal ablation on immunity

**Figure 1:**
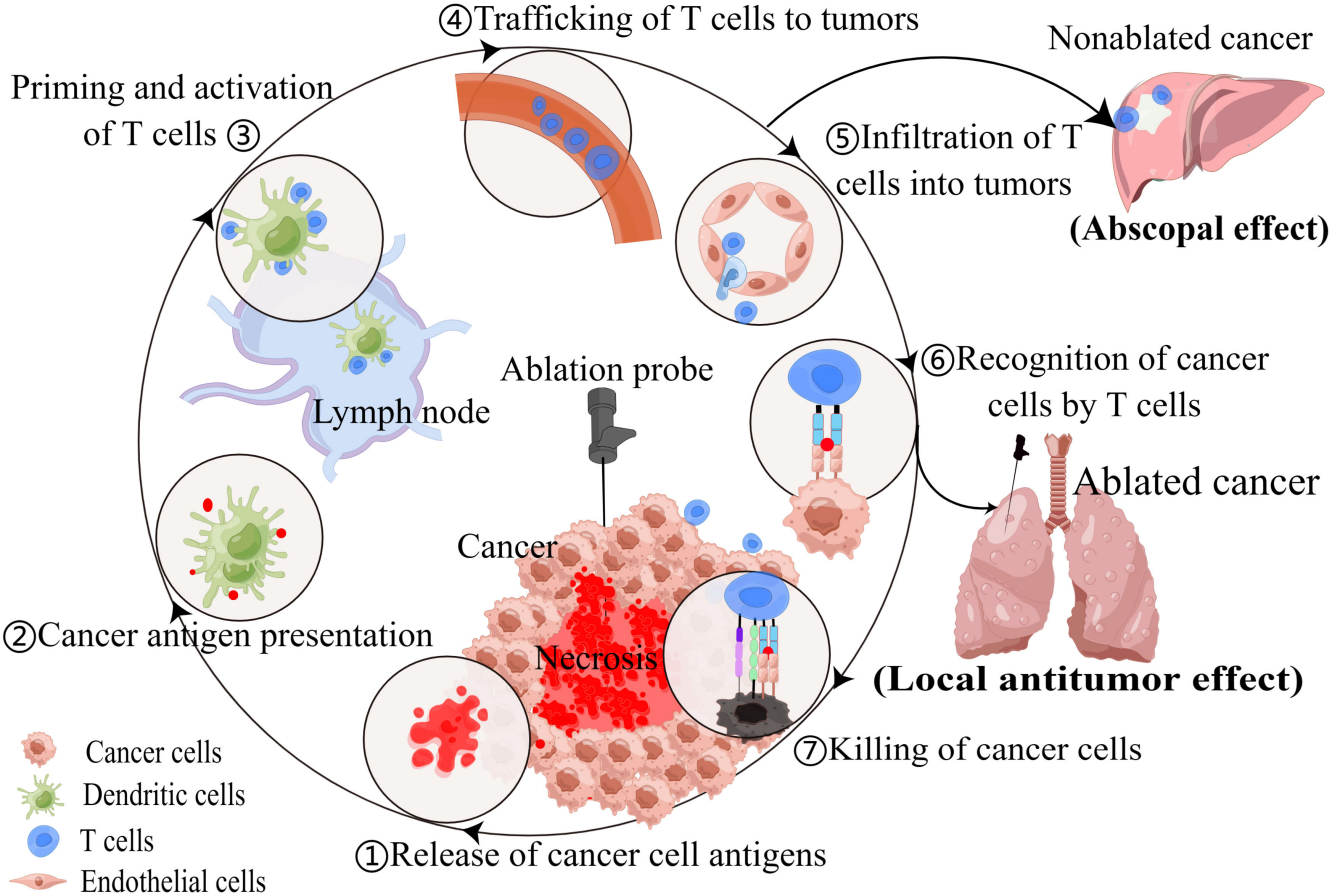
Thermal ablation techniques can modulate anti-tumor immune response, resulting in local antitumor effect and abscopal effect.

The TIME is a critical determinant of tumor progression, metastasis, and response to therapy (27). Thermal ablation techniques can significantly remodel the TIME by inducing immunogenic cell death, releasing cancer antigens, and exposing damage-associated molecular patterns (DAMPs) (12, 28). When cancer antigens are presented to T cells via antigen-presenting cells, such as dendritic cells (DCs), it activates and primes the immune response. This leads to the trafficking and infiltration of cytotoxic T cells into the tumor, resulting in cancer cells death and further antigens release. This process is known as the cancer-immunity cycle (29). For patients with NSCLC, necrotic tumor debris induced by RFA, MWA or cryoablation have the potential to function as *in situ* vaccines that induce autologous antitumor immune responses, including innate and adaptive immunity (12, 30, 31) (**Figure 1**).

In addition, recent studies have highlighted the potential of thermal ablation to induce systemic anti-tumor immunity, exhibiting the abscopal effect in which the ablation of local tumors leads to the regression of distant untreated tumor sites (32–35). Cite a case, a 69-year-old patient with metastatic lung squamous cell carcinoma underwent MWA of a lesion in the right lower lung after developing immunotherapy resistance. Subsequently, tumor shrinkage was observed in the 4R/7 lymph node metastasis (35). This intriguing phenomenon has sparked interest in combining thermal ablation with other immunotherapeutic strategies, such as immune checkpoint inhibitors (ICI), to further enhance anti-tumor immune responses and improve patient outcomes (36, 37). Although the abscopal effect of ablation has been recognized in several cancers, it has been rarely reported in thermal ablation of lung cancer. Perhaps most ablations do not produce abscopal effect, or the effect produced is weak and not clinically manifested, which is closely related to individual differences and ablation techniques.

From above, we know that thermal ablation can induce not only local but also systemic immunity. However, the immune responses induced by different ablation techniques differ due to differences in principles. Compared with hyperthermic ablative methods, cryoablation has been shown to induce a more robust systemic immune response. The basic principle is that thermal ablation denature and degrade the protein, destroying part of the tumor antigen, while cryoablation makes the tumor antigen relatively preserved (29). Erinjeri et al. (38) revealed ablation type was an independent predictor of changes to IL-6 following ablation in human tumors, including lung cancers. Among RFA, MWA, and cryoablation, the latter had the most significant impact on pro-inflammatory cytokine IL-6. This explains to some extent why cryoablation causes strong immune response. In some animal model experiments, MWA appears to induce the weakest immune response (39). However, there seems to be no direct evidence of this phenomenon in lung cancer.

## 3 Biomarkers in lung cancer

### 3.1 Classification of biomarkers

Biomarkers have emerged as invaluable tools in the diagnosis, prognosis, and treatment selection for lung cancer, offering critical insights into the molecular mechanisms underlying the disease. Biomarkers can be classified into four major categories: genomic, proteomic, immunological, and radiological.

Genomic biomarkers: Genomic biomarkers encompass alterations in DNA or RNA sequences, such as mutations, amplifications, deletions, or rearrangements, which can drive tumor progression and influence treatment response (40). A prime example of a genomic biomarker is the epidermal growth factor receptor (EGFR) mutation, which has been associated with targeted therapy response in NSCLC (41). Other notable genomic biomarkers include anaplastic lymphoma kinase (ALK) rearrangements and ROS1 fusions, both of which are also linked to targeted therapy responsiveness in NSCLC (42, 43).

Proteomic biomarkers: The identification of proteomic biomarkers involves the analysis of protein expression and modification, which can aid in the diagnosis, development, and progression monitoring of NSCLC (44). To name just a few examples. The panel of four serum proteins, CEA, CA-125, CYFRA 21-1 and NY-ESO-1 showed good performance in the early detection of NSCLC (45). Zeng et al. (46) demonstrated that a biomarker panel consisting of glutathione S-transferase P1 (GSTP1), heat shock proteinβ-1 (HSPB1), and creatine kinase brain type (CKB) exhibited high sensitivity (92%) and specificity (91%) in distinguishing normal bronchial epithelial tissue, preneoplastic lesions, and invasive lung squamous cell cancer. Hsu et al. (47) observed a positive correlation between ERO1L and NARS levels with lymph node metastasis in lung adenocarcinoma.

Immunological biomarkers: Immunological biomarkers refer to immune-associated molecules, cells, or processes that can affect the anti-tumor immune response and help forecast the reaction to immunotherapy. Examples of these biomarkers include immunoglobulin levels, such as IgM, IgG, and IgA, proteins synthesized by the immune system in response to infection or neoplastic disease (48). Furthermore, programmed death-ligand 1 (PD-L1) expression, tumor mutational burden (TMB), and immune cell infiltration are immunological biomarkers associated with responses to ICI in NSCLC (49).

Radiological biomarkers: Radiological biomarkers refer to imaging features or patterns that can provide information on tumor characteristics, treatment response, or prognosis. Tumor information can be gleaned through diverse imaging techniques like X-ray, computed tomography (CT), magnetic resonance imaging (MRI), and positron emission tomography (PET). Specifically, CT scans or MRIs can elucidate the tumor’s size, its bodily location, and potential spread to adjacent lymph nodes or other organs. PET scans can also help identify areas of increased metabolic activity, which may indicate the presence of cancerous cells. The emerging field of radiomics, which extracts quantitative features from medical images, has shown promise in identifying biomarkers that can predict response to therapy or patient outcomes in lung cancer (50).

### 3.2 The role of biomarkers in lung cancer diagnosis, prognosis, and treatment selection

The identification and validation of biomarkers have substantially transformed lung cancer management in several ways, including diagnosis, prognosis, and treatment selection:

Diagnosis: Biomarkers have displayed immense potential in enabling early lung cancer detection, distinguishing malignant from benign lesions, and identifying specific lung cancer subtypes like NSCLC and small cell lung cancer (SCLC). For example, neuroendocrine markers such as chromogranin, synaptophysin, and CD56 can aid in diagnosing SCLC (51). Additionally, liquid biopsy techniques, like analyzing circulating tumor DNA (ctDNA) and circulating tumor cells (CTCs), are proving invaluable for early detection and diagnosis (52, 53). Recent research also delves into the potential of saliva microbiota and other non-invasive biomarkers for lung cancer detection (54, 55).

Prognosis: Biomarkers can provide essential information on disease aggressiveness, risk of recurrence, or OS, thereby enabling risk stratification and appropriate management decisions. For instance, the detection of specific genomic alterations or high TMB in lung cancer patients has been associated with poorer prognosis (56). Furthermore, DNA methylation biomarkers have demonstrated utility in various aspects of clinical cancer management, including early disease detection, progression and metastasis, treatment, and prognosis (57). Lee et al. (58) proposed that the quantitative CT imaging signature could predicts overall survival in patients with stage I NSCLC.

Treatment selection: Biomarkers play a crucial role in guiding the selection of targeted therapies or immunotherapies, enhancing treatment efficacy and minimizing toxicities. For instance, the presence of EGFR mutations, ALK rearrangements, or high PD-L1 expression can inform the choice of appropriate targeted therapies or immunotherapies for cancer patients (41, 42, 49). TMB in ctDNA shows promise in predicting the effectiveness of PD-L1/PD-1 inhibitors. A study by Kim et al. (59) indicated blood TMB as a potential biomarker for atezolizumab in NSCLC.

## 4 Potential biomarkers for predicting immune response to thermal ablation

### 4.1 Pre-treatment biomarkers

Pre-treatment biomarkers play a crucial role in identifying patients who are most likely to benefit from thermal ablation and in predicting the immune response to treatment. However, there are few studies on these effective markers. Since thermal ablation can induce antigen release followed by immune response and ablation combined with immunomodulation is expected to engender more effective anti-tumor response, markers associated with immune response may also be useful markers for predicting immune response to thermal ablation.

TMB: In recent years, more and more attention has been paid to the relationship between TMB and immunotherapy. High TMB has been linked to increased neoantigen production and enhanced response to immunotherapy (60). Researchers like Samstein et al. (61) have reported that high TMB was associated with improved survival in patients receiving ICI in various malignancies including NSCLCs. Hellmann et al. (62). found that first-line nivolumab combined with ipilimumab in patients with NSCLC and high TMB had longer progression-free survival (PFS) than chemotherapy, regardless of PD-L1 expression level. The results not only validate the benefit of nivolumab in combination with ipilimumab in NSCLC, but also validate the role of TMB as a patient-selected biomarker. Ricciuti et al. (63) found that high TMB levels were associated with increased CD8+, PD-L1+ T-cell infiltration and PD-L1 expression, upregulating innate and adaptive immune response signatures. TMB may be a biomarker for predicting favorable immune responses to thermal ablation. The principle behind this is that tumors with a higher mutation load may release more tumor antigens upon ablation, thus stimulating a more robust anti-tumor immune response.

PD-L1 expression: As the only FDA-approved biomarker for anti-PD-1 therapies, treatments such as cemiplimab-rwlc, pembrolizumab, and atezolizumab may be considered as first-line for patients with advanced/metastatic PD-L1-High NSCLC (63). Despite PD-L1 expression generally being an unfavorable prognostic factor for NSCLC (64), it correlates with an improved response to ICI in lung cancer (65–67). Notably, Rangamuwa et al. (68) observed an increase in tumor PD-L1 expression in NSCLC post bronchoscopic thermal vapor ablation, indicating a potential for a stronger immune response. Hence, we hypothesize that tumors with high pre-treatment PD-L1 expression may elicit a robust immune response following thermal ablation.

Immune cell infiltration: The presence and composition of immune cells, especially tumor-infiltrating lymphocytes (TIL), in the tumor microenvironment can significantly impact the therapy response and prognosis. TIL refers to the heterogeneous lymphocytes in tumor tissue that can specifically kill self-tumor cells and are the mainstay of anti-tumor immune responses (69). Rakaee et al. (70) discovered that a high TIL level (≥250 cells/mm²) was independently associated with a positive response to ICI treatment. Liu et al. (71) reported that the absence of memory B cells or an increased count of M0 macrophages in tumors indicated poor prognosis in early-stage lung adenocarcinoma. They found T follicular helper cells linked to a favorable prognosis in lung squamous cell carcinoma, while increased neutrophil counts suggested the opposite. Kim et al. (72) discovered that high CD3+ T cell infiltration and a low FOXP3+/CD8+ T cell ratio independently predicted a clinical benefit from PD-1 blockade in NSCLC. When combined with MWA, PD-1 blockade treatments like camrelizumab demonstrated improvement in the objective response rate in advanced NSCLC (73). Consequently, immune cells within tumors may emerge as a valuable biomarker predicting the response to PD-1 blockade and thermal ablation and potentially guide therapeutic decisions.

In a review by Darvin et al., the active pursuit of biomarkers to predict responses to ICI was emphasized. Despite these efforts, the research community is yet to definitively identify markers that could accurately select patients with a likely positive response to this new category of therapeutic antibodies (74). This highlights the need for further research in this area to develop better predictive biomarkers for immune response to various treatments, including thermal ablation.

### 4.2 Post-treatment biomarkers

Monitoring the immune response following thermal ablation can provide valuable insights into treatment efficacy and potential immune-related adverse events. Some key post-treatment biomarkers include:

Circulating immune cells: Many studies seeking to understand the impact of thermal ablation on tumor immunity have examined its effects on peripheral blood immune cells, including CD4+ T cells, CD8+ T cells, and DCs. CD4+ T cells are critical elements of effector T cells. After RFA, the shifts in CD4+ T cell subsets in lung cancer patients are diverse. There is an increase in Th1 cells and Th1/Th2 ratio, whereas the levels of Th2, Th17, and regulatory T (Treg) cells decrease. This alteration suggests an enhancement of the anti-tumor immunity (75). Treg cells are immunosuppressive subsets of CD4+ T cells expressing Foxp3, CD25 and CD4, which can inhibit antitumor immune responses in tumor patients (76). To our delight, Fietta et al. (77) notably observed a significant reduction in peripheral blood CD25+Foxp3+ Treg cells 30 days post-RFA in lung cancer patients. These findings align with similar results from a MWA study (78). CD8+ T cells, also known as cytotoxic T lymphocytes (CTL), are primary players in the anti-tumor immune response. Zhang et al. (78) discovered an increased proportion of CD8+ T cells one month after MWA in lung malignancies. A considerable rise in CTL/Treg ratios was also reported after heat-based ablation (79). DCs, serving as specialized antigen-presenting cells, are instrumental in initiating and regulating innate and adaptive immune responses (80). In recent years, much attention has been paid to modulating DCs function to improve cancer immunotherapy. Schneider et al. (31) found that peripheral blood immunostimulatory BDCA-3+/B7-H3-DCs increased in patients with NSCLC after RFA and surgery. Thus, thermal ablation-induced tumor necrosis may act as an *in situ* antigen source to spark an anti-tumor immune response.

Cytokines and soluble factors: Cytokines, soluble low-molecular-weight proteins, secreted by immune cells like lymphocytes, macrophages, and NK cells (81), function as vital mediators in the immune system’s communication network (82). Levels of cytokines and other soluble factors like interferon-gamma (IFN-γ), interleukin-2 (IL-2), or transforming growth factor-beta (TGF-β), can reflect the body’s immune response to diverse stimuli or conditions. IFN-γ, a key coordinator of innate and adaptive immunity (83), exerts an inhibitory effect on primary and metastatic tumors (84). IL-2, a crucial T cell growth factor, was initially used therapeutically to amplify immune responses in cancer patients (85). Xu et al. (86) reported fluctuations in IL-2 and IFN-γ levels in NSCLC patients treated with MWA, with a decrease at 48h post-ablation followed by an increase at 1-month post-ablation. RFA has also been shown to increase levels of IFN-γ in peripheral blood (81). Erinjeri et al. (38) revealed significant post-thermal ablation increases in plasma IL-6 and IL-10 levels in human tumors, including lung cancers. However, IL-1α, IL-2, and TNF-α plasma levels remained unchanged after ablation. Heat shock proteins (HSPs), potent immune system alarms, can trigger antitumor immunity activation. RFA was found to cause HSP70 release into the serum, transiently detectable one day after RFA with over a twofold increase in nine out of 22 cancer patients. Elevated HSP70 serum levels might serve as a biomarker for favorable clinical outcomes (87). The observed differences across various experiments could potentially be attributed to the ablation method and observation time point.

### 4.3 Radiological biomarkers for monitoring the immune response

Radiological biomarkers have emerged as valuable tools for noninvasive monitoring of the immune response, offering crucial insights into treatment outcomes and efficacy. Among the various radiological biomarkers, imaging features and patterns, as well as radiomics, have demonstrated significant potential in this regard.

Imaging features and patterns: Advanced imaging modalities such as dynamic contrast-enhanced MRI (DCE-MRI) and PET have been widely employed in radiological investigations (88). These techniques provide vital information on tumor perfusion, metabolism, and inflammation, which may be correlated with the immune response to various treatments, including thermal ablation and ICI. Alterations in tumor perfusion or metabolic activity following thermal ablation may indicate changes in the TIME, serving as potential indicators of treatment response. Imaging characteristics of the immune response may be different among patients treated with different regimens. Pseudoprogression and hyperprogression are two different response patterns to immunotherapy (89), which are related to changes in the TIME, such as T cell infiltration (90). The term pseudoprogression describes the progression of radiologic tumors in which the tumor size initially increases or new lesions appear followed by a decrease in tumor size or disappearance after some time (91). Although tumor pseudoprogression rarely occurs in lung cancer, many studies have shown that pseudoprogression may ultimately have clinically beneficial (92). Hyperprogression is an atypical response pattern to ICI that manifests as an unexpected radiographic tumor growth (93). It might be associated with effector T cells, Treg cells, macrophages and tumor cells in tumor microenvironment (TME) (94, 95). One study suggested that hyperprogression was more common and a poor prognostic biomarker in NSCLC patients with PD-1/PD-L1 inhibitors (96). Moreover, there is increasing interest in using FDG PET/CT parameters to characterize the TIME and monitor response to ICI (97, 98). Some related metrics, such as metabolic tumor volume (MTV), total lesion glycolysis (TLG) and metabolic activity of the gut microbiome are potential biomarkers of ICI (50). Lower MTV and TLG in patients with NSCLC before treatment were found to have an improved survival (99). Significant reduction of MTV and TLG after 1 month of ICI was related to a better prognosis (100). Cvetkovic et al. (101) revealed that lower colon physiologic 18F-FDG uptake was associated with the response to ICI in patients with advanced NSCLC. These metrics related to ICI may also be the biomarkers of immune response after thermal ablation.

Radiomics: Radiomics is a rapidly evolving field that involves the extraction and analysis of a large number of quantitative features from medical images, offering valuable diagnostic, prognostic or predictive information (102). By identifying unique imaging patterns and analyzing various features, including tumor shape, size, texture, and intensity, radiomics can reveal associations between imaging characteristics and the underlying TIME (103). A machine learning approach was used by Tong et al. (104) to predict TIME profiles in NSCLC based on the radiomics and clinical characteristics of 18F-FDG PET/CT scans. Results showed that 18F-FDG PET/CT radiomics combined with a clinical model was a clinically practical approach to noninvasively detecting tumor immunity in NSCLCs. Radiomic markers extracted from baseline CT images of advanced NSCLC patients treated with PD-1/PD-L1 inhibitors may assist in predicting hyperprogression in one study (105). Similarly, another study found that radiomic texture changes (“delta”) could predict response to ICI therapy and OS for patients with NSCLC (106). Sun et al. (107) have successfully defined and validated a radiomics signature for predicting intratumoral CD8 T cells infiltration (CD8 rich or CD8 poor), which included 8 variables. Similarly, Jiang et al. (108) explored different radiomic-based predictive models to access PD-L1 expression level. Finally, it was confirmed that the CT-derived prediction model could predict the PD-L1 expression status of NSCLC patients relatively accurately. Radiomics using deep learning can further characterize tumors, it may be a valuable tool for monitoring the immune response to thermal ablation and predicting treatment outcomes in lung cancer patients.

**Figure 2:**
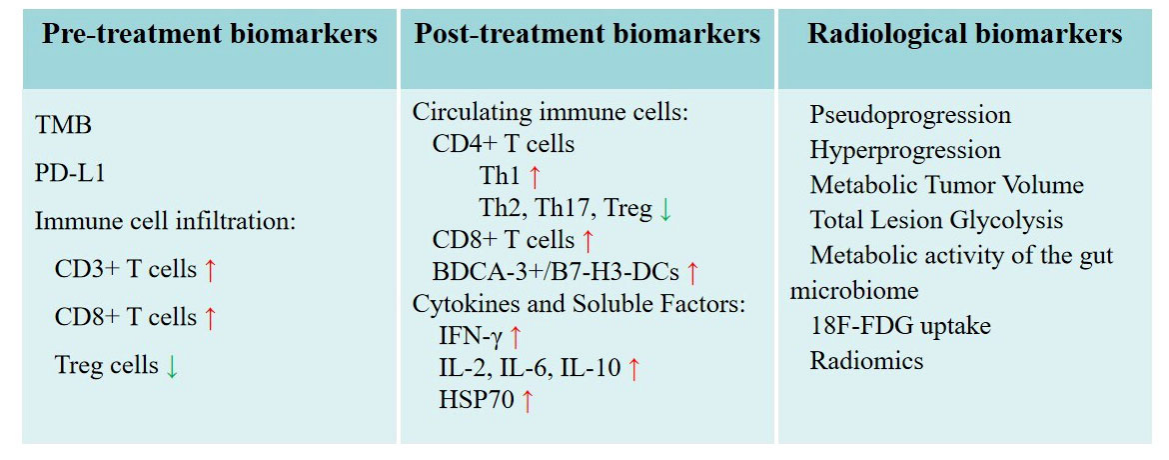
Potential biomarkers for predicting immune response to thermal ablation. Radiological biomarkers as special biomarkers are listed separately.

A summary of biomarkers for predicting immune response to thermal ablation are presented in **Figure 2**.

## 5 Potential biomarkers for predicting outcomes in lung cancer patients undergoing thermal ablation

**Figure 3:**
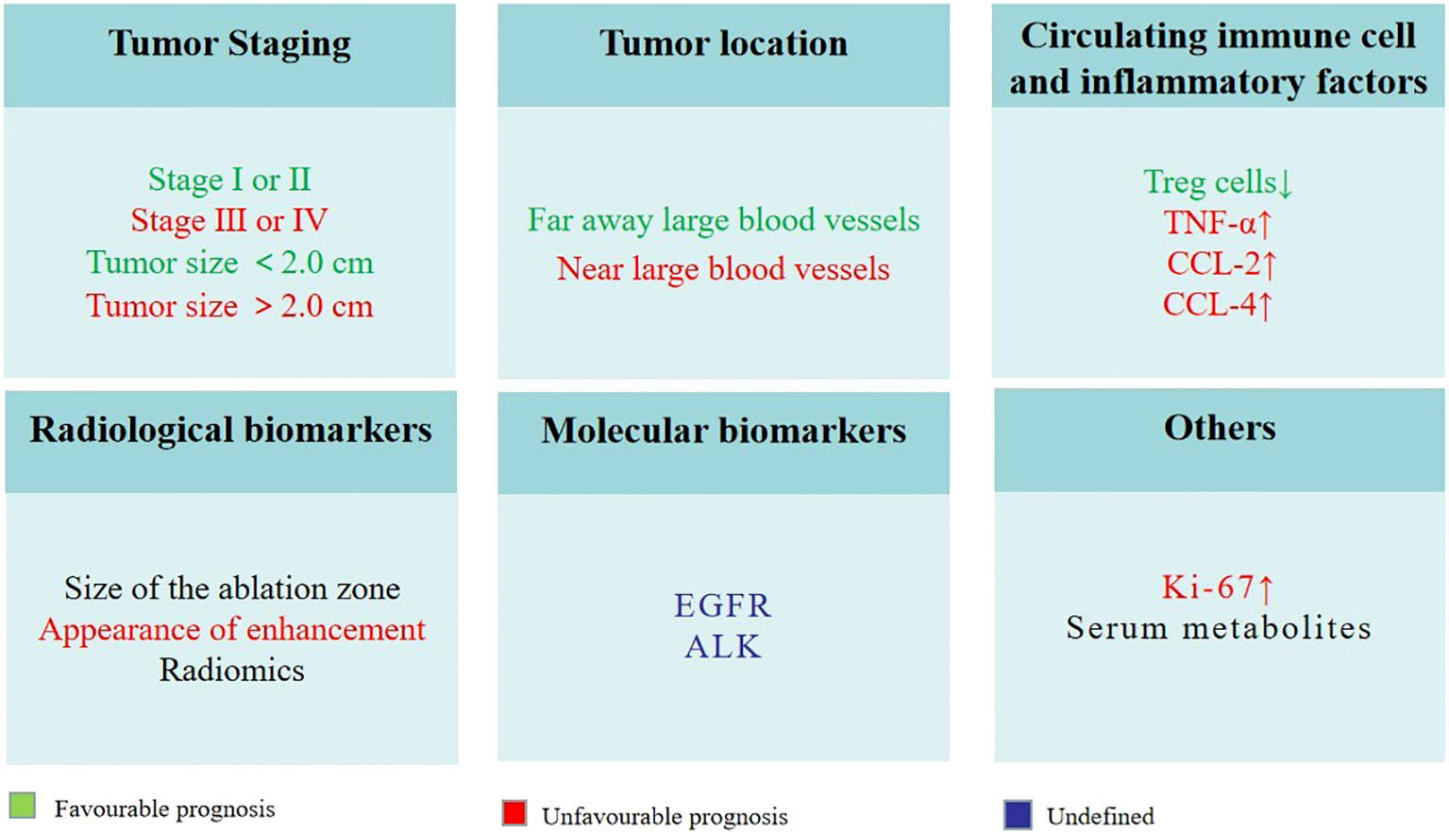
Potential biomarkers for predicting outcomes in lung cancer patients undergoing thermal ablation.

Biomarkers associated with local tumor control and recurrence, OS and PFS play a pivotal role in selecting appropriate candidates for thermal ablation and identifying patients who are likely to exhibit better long-term outcomes. Several potential biomarkers have been identified in the literature (**Figure 3**).

a) Tumor staging: Early-stage lung cancer patients (e.g., stage I or II) have been shown to exhibit superior OS and PFS following thermal ablation as compared to patients with advanced-stage disease. In the Das et al. study, median PFS and OS in stage IIIB or IV NSCLC patients treated with MWA were 11 months and 18 months, respectively (109). However, in the Nance et al. study, median PFS and OS in Stage I NSCLC patients treated with MWA were 19.1 months and 26.2 months, respectively (110). Tumor size is an important part of TNM stage. A number of studies have demonstrated that smaller tumor size is associated with better local control and lower recurrence rates following thermal ablation in lung cancer patients (111). For instance, a study by Dupuy et al. (111) found that NSCLC patients receiving RFA treatment with tumors smaller than 2.0 cm had a significantly better prognosis than those with larger tumors. Thereby, early detection and appropriate staging of lung cancer are crucial in determining the best treatment strategy and improving patient outcomes.

b) Tumor location: The location of the tumor and its proximity to critical structures can influence the success of thermal ablation and the risk of recurrence. Capillary perfusion and blood flow to large vessels within the tissue can significantly reduce ablation extent (112), patients with tumors far away these locations were more likely to have a favorable response to treatment and a reduced risk of recurrence.

c) Circulating immune cell and inflammatory factors: Peripheral blood immune cells and inflammatory factors can affect local tumor control, recurrence and survival. A recent study found that reduced Treg cells were independently associated with PFS after MWA in patients with pulmonary malignancies (78). Median PFS times for patients with higher Treg cell reductions were significantly longer than those with lower reductions (16 months vs. 8.5 months, p = 0.025). Schneider et al. (113) studied the changes of tumor necrosis factor (TNF)-α, chemokine (C-C motif) ligand (CCL)-2 and CCL-4 of patients with NSCLC. Compared to patients without relapse, the researchers found a significant increase in both CCL-2 and CCL-4 levels early in patients with local or lymphogenic tumor relapses. The increased production of NO by myeloid-derived suppressor cells (MDSC) may contribute to these changes. It might be an early indicator of the incomplete RFA and subsequently a potential tumor relapse in NSCLC. There is a possibility that it could be an early indication of the incomplete RFA and tumor relapse in NSCLC.

d) Radiological biomarkers: Using contrast-enhanced CT, PET/CT or cone-beam CT (CBCT) for imaging follow-up is very important for supervise residual or recurrent lung disease after thermal ablation. It is highly likely that incomplete ablation will occur if there is no complete encirclement of the tumor during postoperative CBCT (114). At the 1-month post-treatment evaluation, the presence of an incomplete ablation can be inferred if there is no observed enlargement in the ablation zone or if the consolidation exhibits nodular enhancement resembling the characteristics of the initial tumor (115). The presence of any increase in the dimensions of the ablation region at the 6-month mark indicates a potential recurrence (115). Throughout the entire follow-up process, the presence of central or peripheral nodular or irregular enhancement should be regarded as indicative of residual or recurrent disease (115, 116). Radiomics with high-throughput features have been explored for various clinical applications including lung cancer. Liu et al. (117) retrospectively observed the instantaneous changes in intratumor density heterogeneity after MWA of lung tumors via radiomics features. They found significant correlations between the visual score of ablation response and quantitative features. Changes in local features after MWA had the most significant correlation. More importantly, thy revealed △contrast% was a better predictor of 1-year local tumor progression. Radiomics, including clinical, radiological, and technical features, have also been explored in predicting local tumor progression of colorectal cancer lung metastases treated with RFA (118).

e) Molecular biomarkers: Certain molecular biomarkers, such as EGFR and ALK, are used for predicting the response to chemotherapy and targeted treatments in lung cancer patients (119, 120). However, there is limited information available on the role of these biomarkers in predicting treatment outcomes for patients undergoing thermal ablation. Further research is needed to determine the potential of molecular biomarkers in predicting the success of thermal ablation and the risk of recurrence in lung cancer patients.

f) Others: One study performed histopathological analysis of tissue extracted from electrodes after RFA of lung tumors. The results showed that the presence of Ki-67+ cells in the tumor after ablation was associated with a 3-fold increased risk of death from cancer and local tumor progression (121). It is easy to understand and accept this result, because Ki-67 as a proliferation index, its appearance represents incomplete ablation, so the tumor is prone to recurrence and affects the prognosis. However, we were inspired by this finding that detection of Ki-67 in the ablated tumor has certain clinical significance. 1H nuclear magnetic resonance (NMR)-based metabolomics analysis was employed to find potential serum biomarkers of MWA, Hu et al. (122) found that serum lactate, alanine and glutamate levels were increased significantly, while serum glucose, taurine and glutamine levels were decreased. A disturbance in serum metabolites has been proposed to be a potential biomarker for MWA efficacy in NSCLC therapy.

It is important to note that these biomarkers should be considered in combination and as part of a multimodal approach for predicting outcomes in lung cancer patients undergoing thermal ablation. More research in tumor and host immune-specific factors may identify additional biomarkers that can better predict response and prognosis in these patients.

## 6 Potential confounding factors and challenges in the identification and validation of predictive biomarkers

The process of identifying and validating predictive biomarkers for immune response and outcomes in lung cancer patients undergoing thermal ablation is riddled with complexities and obstacles due to several factors:

Ablation techniques: As previously mentioned, the effects of different ablation techniques on the body’s immunity are diverse. Besides, other factors such as ablation parameters, observation time points, and individual differences can lead to biased observations. Biomarkers may be influenced by those factors.

Heterogeneity: Lung cancer is an extremely heterogeneous disease, encompassing multiple subtypes and diverse molecular profiles. TIME is also different in different lung cancers, it can be roughly divided into three different immunophenotypes (123). This heterogeneity complicates the task of pinpointing and validating biomarkers that can be universally applied across various patient populations.

Complex tumor-immune interactions: The dynamic and intricate interplay between tumors and the immune system has a profound impact on treatment responses and outcomes. Gaining a comprehensive understanding of these complex interactions and identifying reliable biomarkers that can accurately predict outcomes remain formidable challenges.

Limited sample size and retrospective studies: A significant number of studies investigating predictive biomarkers in thermal ablation are characterized by small sample sizes or rely on retrospective data, which may hinder the generalizability of their findings. This limitation makes it difficult to establish robust and conclusive associations between biomarkers and treatment outcomes.

Confounding factors and biases: Bias and confounding factors can impact the validity of identified biomarkers, potentially leading to inaccurate conclusions. A comprehensive evaluation of these factors is crucial in order to ensure the reliability of the identified biomarkers and their potential clinical application.

To address these challenges and pave the way for the successful identification and validation of predictive biomarkers in lung cancer patients undergoing thermal ablation, researchers must adopt a multifaceted approach that includes:

Large-scale, prospective studies: Conducting well-designed, prospective studies with large sample sizes can help to overcome the limitations associated with small sample sizes and retrospective data, thereby improving the generalizability of the findings and facilitating the discovery of robust biomarkers.

Addressing heterogeneity: Researchers should consider the diverse molecular profiles and subtypes of lung cancer when investigating potential biomarkers. This may involve stratifying patient populations based on specific molecular features or subtypes (124), thereby increasing the likelihood of identifying reliable and clinically relevant biomarkers.

Controlling for confounding factors and biases: Employing robust statistical methods, such as matching or adjusting for potential confounding variables, can help to minimize the impact of confounding factors and biases on the study results (125). This will enable more accurate identification and validation of predictive biomarkers.

Integrating multidisciplinary expertise: Collaborations between experts in various fields, such as oncology, immunology, and bioinformatics, can facilitate a more comprehensive understanding of the complex tumor-immune interactions and the identification of reliable biomarkers. This multidisciplinary approach can help to overcome some of the challenges associated with the identification and validation of predictive biomarkers in the context of lung cancer and thermal ablation.

Leveraging advanced technologies: Employing cutting-edge technologies, such as next-generation sequencing and high-throughput screening, can expedite the discovery and validation of predictive biomarkers (126). These technologies can provide a wealth of data, which, when combined with robust statistical analyses, can help to unravel the complex relationships between biomarkers and outcomes in lung cancer patients undergoing thermal ablation.

## 7 Future directions and clinical implications

### 7.1 The potential of combining biomarkers for improved prediction accuracy

Combining multiple biomarkers may improve the accuracy of predicting treatment outcomes in lung cancer patients undergoing thermal ablation. By considering various factors, such as tumor characteristics, molecular markers, and immune cell infiltration, clinicians can develop a more comprehensive understanding of each patient’s individual disease biology and tailor treatment strategies accordingly. Future research should focus on identifying and validating biomarker combinations that provide the greatest prognostic value for patients undergoing thermal ablation.

### 7.2 Integrating biomarker analysis into clinical decision-making and treatment planning

The integration of biomarker analysis into clinical decision-making and treatment planning can help optimize patient outcomes and minimize treatment-related toxicity. For example, clinicians can use biomarkers to select patients who are most likely to benefit from thermal ablation or to identify those at risk of complications or recurrence. Additionally, monitoring the immune response to treatment using post-treatment biomarkers can help guide the use of adjuvant therapies, such as immunotherapy or targeted therapy, to enhance treatment efficacy and prolong survival. Further research is needed to develop standardized guidelines and protocols for the integration of biomarker analysis in clinical practice.

### 7.3 The role of emerging technologies

Emerging technologies, such as liquid biopsies and artificial intelligence (AI), hold promise for advancing biomarker discovery and validation in lung cancer patients undergoing thermal ablation. Liquid biopsies, which involve the analysis of ctDNA or CTCs in blood samples, can provide real-time information on tumor molecular characteristics and treatment response. This noninvasive approach may facilitate the identification of new biomarkers that can be used to monitor the immune response and predict treatment outcomes. AI can be used to analyze large, complex datasets and identify patterns that may be missed by traditional statistical methods. For example, machine learning algorithms can be applied to radiomics data to uncover novel imaging biomarkers that correlate with the immune response to thermal ablation. Additionally, AI can be used to integrate various types of biomarker data, such as genomic, proteomic, and radiological data, to create more accurate predictive models for treatment outcomes.

In conclusion, the future of biomarker research in lung cancer patients undergoing thermal ablation is likely to be driven by advances in technology and the integration of multiple biomarker modalities. This will ultimately enable clinicians to develop personalized treatment strategies that maximize patient outcomes and minimize treatment-related toxicity.

## 8 Conclusion

This review highlighted the importance of various biomarkers for predicting immune response and outcomes in lung cancer patients undergoing thermal ablation. The integration of biomarker analysis into clinical practice has the potential to significantly impact personalized treatment strategies and improve patient outcomes. By identifying patients who are most likely to benefit from thermal ablation, as well as those at risk of complications or recurrence, clinicians can tailor treatment approaches to each individual patient’s unique disease biology. Furthermore, the use of biomarkers to monitor the immune response to treatment can help guide the use of adjuvant therapies, such as immunotherapy or targeted therapy, to enhance treatment efficacy and prolong survival.

## Author contributions

JS: Writing – original draft. XY: Writing – review & editing.
